# Exploring a Need for a Cardiometabolic Disease Staging System as a Computerized Clinical Decision Support Tool: Qualitative Study

**DOI:** 10.2196/37456

**Published:** 2022-07-01

**Authors:** Aizhan Karabukayeva, Jami L Anderson, Allyson G Hall, Sue S Feldman, Tapan Mehta

**Affiliations:** 1 Department of Health Services Administration School of Health Professions University of Alabama at Birmingham Birmingham, AL United States; 2 Department of Graduate Medical Education Heersink School of Medicine University of Alabama at Birmingham Birmingham, AL United States; 3 Department of Family and Community Medicine Heersink School of Medicine University of Alabama at Birmingham Birmingham, AL United States

**Keywords:** cardiometabolic disease staging system, risk assessment, cardiometabolic disease, clinical decision support system, primary care, obesity, overweight, medical management

## Abstract

**Background:**

Although cardiometabolic diseases are leading causes of morbidity and mortality in the United States, computerized tools for risk assessment of cardiometabolic disease are rarely integral components of primary care practice. Embedding cardiometabolic disease staging systems (CMDS) into computerized clinical decision support systems (CDSS) may assist with identifying and treating patients at greatest risk for developing cardiometabolic disease.

**Objective:**

This study aimed to explore the current approach to medical management of obesity and the need for CMDS designed to aid medical management of people living with obesity, at risk of being obese, or diabetic at the point of care.

**Methods:**

Using a general inductive approach, this qualitative research study was guided by an interpretive epistemology. The method included semistructured, in-depth interviews with primary care providers (PCPs) from university-based community health clinics. The literature informed the interview protocol and included questions on PCPs’ experiences and the need for a tool to improve their ability to manage and prevent complications from overweight and obesity.

**Results:**

PCPs (N=10) described their current approaches and emphasized behavioral treatments consisting of combined diet, physical activity, and behavior therapy as the first line of treatment for people who were overweight or obese. Results suggest that beneficial features of CDSS include (1) clinically relevant and customizable support, (2) provision of a comprehensive medical summary with trends, (3) availability of patient education materials and community resources, and (4) simplicity and ease of navigation.

**Conclusions:**

Implementation of a CMDS via a CDSS could enable PCPs to conduct comprehensive cardiometabolic disease risk assessments, supporting clinical management of overweight, obesity, and diabetes. Results from this study provide unique insights to developers and researchers by identifying areas for design optimization, improved end user experience, and successful adoption of the CDSS.

## Introduction

Cardiometabolic diseases are leading causes of morbidity and mortality in the United States, including a wide array of diseases, typically beginning with insulin resistance and progressing later into a cluster of conditions that increase the risk of type 2 diabetes, stroke, and cardiovascular disease [1,2]. Being overweight (BMI ≥25 kg/m^2^) is associated with double the risk of developing cardiometabolic multimorbidity, while having mild and severe obesity (BMI ≥30 kg/m^2^) increases the risk 4 and 10 times, respectively [3]. However, current diagnostic categories that are based on standard BMI ranges defining overweight and obesity have high specificity but low sensitivity for identifying insulin resistance and cardiometabolic disease [4]. For example, with the current diagnostic categories, some individuals with overweight and obesity might not have cardiometabolic risk factors and may exhibit low rates of future diabetes and cardiovascular-related mortality; alternatively, some individuals who do not meet criteria for either metabolic syndrome or prediabetes exhibit risk of future diabetes [4]. Thus, risk assessments for cardiometabolic disease with greater sensitivity should be an integral component of medical practice, with tools to evaluate preventive and therapeutic options in patients at greatest risk for developing disease. Currently, there is no stratification of the population by level of obesity-related disease and mortality risk [5].

An estimated 42.5% of US adults aged 20 years and older are living with obesity, including 9.0% with severe obesity, and another 31.1% are overweight [6]. Because this group is at high risk of developing diabetes and other obesity-related complications, there is a need for risk stratification approaches to identify early those at highest risk and identify weight loss programs with appropriate treatment intensity. To provide appropriate medical management of obesity and facilitate the diabetes risk assessment of people with excess adiposity, a comprehensive staging system that establishes 5 stages of cardiometabolic disease risk—the cardiometabolic disease staging system (CMDS)—was developed [7,8]. This validated staging system is based on Adult Treatment Panel III metabolic syndrome risk factors and includes waist circumference, systolic and diastolic blood pressures, fasting and 2-hour blood glucose levels, triglycerides, and high-density lipoprotein cholesterol (HDL-C; Table 1) [4]. The purpose of this system is to help clinicians select treatment modality and intensity in the management of cardiometabolic diseases while balancing benefit and risk. Evidence demonstrates the CMDS has higher predictive and discriminative ability compared with other systems and relies on data typically collected during primary care visits; thus, it is more feasible to integrate into busy workflows of primary care providers (PCPs) [5].

**Table 1:** The cardiometabolic disease staging (CMDS) system.

Stage	Descriptor	Criteria
Stage 0	Metabolically healthy	No risk factors
Stage 1	One or two risk factors	Have 1 or 2 of the following risk factors: High waist circumference (≥112 cm in men and ≥88 cm in women) Elevated blood pressure (systolic ≥130 mm Hg and/or diastolic ≥85 mm Hg) or on antihypertensive medication Reduced serum HDL-C^a^ (<1.0 mmol/L or 40 mg/dL in men; <1.3 mmol/L or 50 mg/dL in women) or on medication Elevated fasting serum triglycerides (≥1.7 mmol/L or 150 mg/dL) or on medication
Stage 2	Metabolic syndrome or prediabetes	Have only 1 of the following 3 conditions in isolation: Metabolic syndrome based on 3 or more of 4 risk factors: high waist circumference, elevated blood pressure, reduced HDL-C, and elevated triglycerides Impaired fasting glucose (IFG; fasting glucose ≥5.6 mmol/L or 100 mg/dL) Impaired glucose tolerance (IGT; 2-h glucose ≥7.8 mmol/L or 140 mg/dL)
Stage 3	Metabolic syndrome + prediabetes	Have any 2 of the following 3 conditions: Metabolic syndrome IFG IGT
Stage 4	T2DM^b^ and/or CVD^c^	Have T2DM and/or CVD: T2DM (fasting glucose ≥126 mg/dL or 2-h glucose ≥200 mg/dL or on antidiabetic therapy) Active CVD (angina pectoris or status post a CVD event such as acute coronary artery syndrome, stent placement, coronary artery bypass, thrombotic stroke, nontraumatic amputation due to peripheral vascular disease)

^a^HDL-C: high-density lipoprotein cholesterol.

^b^T2DM: type 2 diabetes mellitus.

^c^CVD: cardiovascular disease.

Vigilance in the management of modifiable risk factors is critical, given that people with overweight and obesity are at increased cardiovascular risk. Primary care settings, as familiar and accessible clinical venues for patients, are well positioned to screen people with overweight and obesity and recommend appropriate weight loss treatment plans to prevent complications and weight progression. Many studies found that the largest weight losses were achieved with high-intensity counseling by PCPs and referral of interested individuals to appropriate interventions [9-13]. However, a study of a nationally representative sample of adults aged 35 years and older found that, despite more adults reported being screened for obesity (78.6%) and of those screened, nearly 40% had a BMI of 30 kg/m^2^ or higher (39.2%), only slightly more than one-half (53.5%) of obese adults screened reported receiving counseling about weight management [14]. Furthermore, BMI is the most preferred screening tool, though literature indicates it could be a poor indicator of cardiovascular disease and overall mortality risk [7,15]. Research finds BMI is not a good index of visceral fat, which is the basis of metabolic disorders associated with increased cardiovascular risk, whereas waist circumference might be superior as a risk assessment tool [16]. PCP-indicated practice improvements, helpful in treating and managing overweight and obesity, include better tools for early identification of risk and preventive treatment for those with multiple risk factors [11].

Providing CMDS to PCPs via computerized clinical decision support systems (CDSS) may assist in stratifying the population by obesity-related disease risk and targeting those patients who are at greater risk for obesity-related complications. To the authors’ knowledge, this would be the first electronic health record–integrated CDSS that would incorporate CMDS. Despite literature indicating CDSS may have a positive impact on provider performance and patient outcomes [17], evidence also indicates that CDSS rarely reach their full potential [18]. As with any innovation, user acceptance and integration within the clinical workflow are critical for successful uptake and routine use [19].

**Figure 1:**
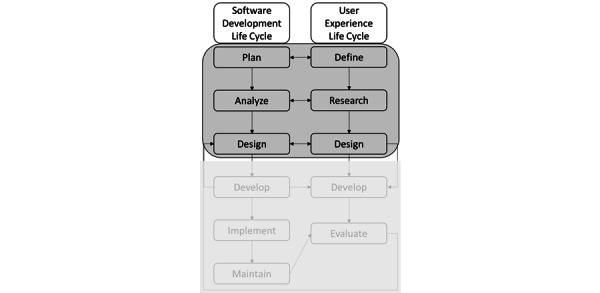
Conceptual model for system analysis and design.

System analysis and design involve the process of planning, analyzing, designing, developing, implementing, and maintaining systems. A user-centered approach focusing on the user experience necessitates coordinated relationships between the system specialists, designers, and developers and the nonspecialists and users with outcomes knowledge. The system development life cycle, when combined with the user experience life cycle, allows for that coordination to occur and has been shown to lead to better system adoption [20]. Figure 1 illustrates our conceptual model for system analysis and design of the CMDS. This paper reports on the first 3 phases of each cycle: (1) plan and define, (2) analyze and research, and (3) design. As such, with the aim of involving users at key milestone stages of system development, this study explored the current approach to management of overweight and obesity and a need for the CMDS system at the point of care to facilitate specificity in treatment modalities. 

## Methods

To ensure we adhered to qualitative reporting standards, we followed the 32-time consolidated criteria for reporting qualitative studies (COREQ) checklist (Multimedia Appendix 1).

### Sampling

Participant recruitment used convenience sampling where the research team coordinated with the medical director for primary care of a large academic medical center in the southeastern United States. Recruitment emails to potential candidates indicated the study purpose and invited participation. The number of participants was determined to be sufficient when saturation was reached (N=10) [21]. All participants were provided with a US $100 gift card. The present study is a foundation for ongoing research aimed at developing and implementing a CDSS based on the CMDS.

### Data Collection

From August 2020 to January 2021, 10 semistructured interviews were conducted by 2 research team members, consisting of the principal investigator (TM; male) and a graduate research assistant (AK; female). Two senior female researchers (AH and SF) with training in qualitative interviewing provided guidance and supervision. The interviewers did not have prior relationships with the participants. Only the interviewers and participants were present during data collection. The duration of the interviews varied between 30 minutes and 45 minutes and were conducted via a collaborative, cloud-based videoconferencing service at a mutually agreed-upon time. Interviews were transcribed verbatim by a commercial transcription company. The interview guide was informed by the literature review and included questions designed to (1) understand how PCPs manage overweight and obesity and facilitate prevention and management of diabetes and cardiovascular disease risk during a standard primary care visit and (2) explore PCP needs for CMDS and preferences for a CDSS (Multimedia Appendix 2). Broad, open-ended questions along with permissive prompts were used to facilitate each semistructured interview. Prior to conducting interviews, the semistructured interview guide was pilot tested with several providers to ensure questions were clear, generated in-depth discussion, were acceptable to participants, and resulted in usable information. Feedback from pilot testing was used to modify the wording, content, and order of the interview questions.

### Ethical Considerations

All investigations were conducted in conformity with ethical principles of research. Consent for participation and interview recording was obtained verbally before each interview. This study was determined to be exempt by the University of Alabama at Birmingham Institutional Review Board (IRB Protocol Number 300003559).

### Data Analysis

Transcribed interviews were coded using an inductive thematic analysis approach with NVivo 12 Plus (QSR International, Melbourne, Australia). To increase reliability and reduce bias, all transcripts were coded by 2 team members (AK and JA) with expertise in thematic analysis [22,23]. The analysis consisted of 2 phases: codebook development and codebook refinement. First, during open coding, coders examined an initial set of transcripts for categories (processes or events that share an attribute) of information related to our research questions. The second phase of our analysis focused on comparing and applying our initial codes to both existing and new data generated from subsequent interviews. This constant comparative analysis [24] across data sets allowed merging and clarifying codes. Following the initial coding process, research team members (AK and JA) discussed questions and discrepancies until 95% agreement was reached. Then, coders identified key points and recurring categories and themes that were central to the experience described by the participants. The process consisted of both coders dividing the text into semantic segments, labelling the segments with codes, together examining the codes for overlap and redundancy, and aggregating these codes into broader categories and themes [25].

## Results

### Sample Characteristics and Suggestions

We recruited 10 PCPs (7 physicians and 3 certified registered nurse practitioners) with practice experience ranging from 3 years to 43 years, with a mean of 12.2 years. Out of 10 respondents, 4 were male, and 6 were female. The most common practice-based barriers included lack of time and knowledge of resources, including access to evidence-based medical models and affordable community options. Considering the results of this study, 4 factors emerged as important for consideration in the development of a CDSS for metabolic conditions: (1) clinically relevant and customizable information delivery, (2) provision of comprehensive medical summary with trends, (3) availability of patient education materials and community resources, and (4) simplicity and ease of navigation. Table 2 describes the key suggestions voiced by the PCPs for future design of the CDSS to be successfully adopted.

**Table 2:** Suggestions from primary care providers regarding preferrable clinical decision support system features.

Suggestions	Quotes
Speed of the information technology	“The other thing would be – does it run efficient? There are parts of Cerner that literally if you click the button, you’re going be sitting there for 2 minutes just waiting, waiting, and waiting.” [Primary care physician, male]
Synthesis of available information	“I think what would be good is if you had a piece of software that could extract that [lab] data out of the record. And then you could click on a button at the top of the record, and it said ‘weight management’. If you click, it would have drop down algorithm and it was connected to the orders.” [Primary care physician, male]
Fit in the workflow	“So, whatever you come up with has to be something that’s integrated and uses the data that’s there, and gives you immediate feedback. It can’t be something that takes three minutes to enter the data.” [Primary care physician, male]
User-friendly with minimalist design	“So, ideally something self-contained, within the same page gives me kind of risk information and recommendations based of that, especially if it could be set up such that off of that page, I could directly order things. That would be amazing.” [Primary care physician, female]
Flexibility	“I think you definitely need to maintain the ability to customize or edit because, again, these are just sort of recommendations and sort of a part of the picture that the risk calculator gives you, but, you know, as long as you know, you could sort of edit to customize and individualize to a patient.” [Primary care physician, female]
Justification of treatment based on guidelines	“If there was something to standardize [management of] obesity and would give you a quantifiable number that puts them at a higher risk factor. So, if there was something that took in more either genetic versus biological markers that could be influential, I think that would be very useful and something that we would definitely want to implement and make it more of a standardization and not just an extra research tool.” [Primary care physician, male]

### Current Management Practice of Obesity in PCP Clinics

#### Focus Not on Prevention But on Comorbidities

Almost all respondents reported that a significant portion of their patient populations was overweight, and they also noted that about 60% to 70% of patients had hypertension, diabetes, or other comorbidities. Even young populations presenting to primary care tended to have elevated BMIs or abnormal glucose levels. However, the respondents noted that they gave priority to management of the comorbidities rather than focusing on prevention and management of obesity. Respondents also noted they did not routinely use pharmacologic treatments for overweight or obesity but more to treat comorbidities, such as hypertension or elevated blood glucose levels.

About 80% of the patients I see are going to have one of the three: hypertension, diabetes, or obesity; and probably safe to say 60-70% definitely have all three. A lot of what I'm seeing is for blood pressure and diabetes management specifically.

Certified registered nurse practitioner, female

I try not to [prescribe medicine]. I have a fair number of patients that would like a pill to fix their weight problem. And sometimes they break me down and I do [prescribe medicine]. If I prescribe something, an appetite suppressant to help lose weight, it's under the premise that it's very short term, no more than three months.

Internist, female

Probably due to the acuity of our patient population, I feel like by the time people get to us specifically in the health system we're working under, there are usually a lot more problems that we're juggling, and obesity is always important, but it’s probably like number 10 on the list of concern.

Certified registered nurse practitioner, male

#### BMI as a Main Diagnostic Measure

According to the respondents, BMI remains the primary tool for assessing obesity, as it is easy to access, is affordable to measure, and can conveniently be used to monitor weight changes. Additionally, participants responded that waist circumference measurement has not been integrated into routine practice. Patient risk factors associated with being identified or diagnosed as overweight or obese by their physician included higher BMI, family history, lifestyle, and habits. Respondents noted that they provided metabolic screening depending on patient’s BMI, including blood glucose and blood lipids levels.

We don't measure it [waist circumference] in our clinic. We do have the BMI. So, the first point is BMI- this is all I look at because that's what I have available, and it's just a measure of numbers and the calculation. So, it's easy.

Internist, female

I look at their medical and family history. Like if they have diabetes in the family, then obviously that puts them at a higher risk automatically. Or if they have family members with hypertension. So, family history is very important for my understanding.

Internist, female

#### Reliance on Lifestyle Modifications

Most of the respondents’ approaches to weight management were limited to assessing physical activity and assessing readiness for change, dietary habits, and expectations. The most common recommendations were to increase physical activity and dietary changes. Interestingly, half (5/10, 50%) of the respondents noted they did not have any formalized treatment plan to manage overweight or obesity and did not follow specific treatment guidelines. In addition, there was limited use of external sources of weight management support, with only few patients being referred to weight loss clinics, mainly due to limited coverage of services by health insurance companies. External resources frequently included a nutritionist and a commercial weight loss program (eg, Weight Watchers).

I do not have sort of very specific treatment guidelines. I’m not saying I’d be opposed to that. I just have my own practice at this time. It really will depend, because I’m trying to gauge a person’s willingness to change, so I will certainly ask some typical, open-ended questions about what have they tried in the past. It really becomes an individualized approach.

Internist, male

I would say exclusively exercise and diet. More recently, I [started] referring patients with BMI 30 or higher with multiple comorbidities to the weight loss management program.

Primary care physician, female

#### Lack of Knowledge About Referral Options in the Community

Respondents agreed that resources for intense lifestyle intervention and social support were important for the patients; however, respondents also noted the lack of knowledge about referral options in the community, including commercial-based programs. Because of the range in the socioeconomic status of their patient population, respondents expressed wanting point-of-care information about various affordable and convenient options that would be readily accessible and affordable for patient engagement.

I think there are a lot of resources out there but to be honest with you, I don’t think that we really know where the resources are. Everyone, probably, has their own little list of resources that they use, I think.

Primary care provider, male

I don’t think I necessarily have a good handle on it [the local resources]. I have certain things that I would say as a ‘go to’ that are probably out of date and missing a lot of some of the newer [resources].

Certified registered nurse practitioner, female

#### Lack of Patient Education Literature

One of the challenges voiced by respondents was lack of appropriate, “meet them where they are” weight management educational materials accessible for use at the point of care or after consultation.

Time during a visit is at a premium. In theory, our visits are 20 minutes, by the time the patient gets here, checks in, and triaged, I generally have about seven minutes out of 20 minutes to see a patient. Maybe a little bit more, sometimes a little less.

Primary care physician, male

The printed materials are not very good that we have available. They are not very helpful. That is why I don’t give them out very often.

Primary care physician, female

### Need for CMDS

#### Need for a Risk Stratification Tool Embedded Into the CDSS

Almost all respondents (9/10, 90%) expressed an interest in having a CDSS that would incorporate diabetes and cardiovascular disease risk assessment and, based on the risks, outline a treatment plan. As respondents noted, the advantage of using a CDSS would be providing “legitimate justification” for a treatment plan with an assumption that patients understand their risk and the reason for the proposed treatment. To the authors’ knowledge, there is no decision support system available to assist providers in evidence-based weight loss treatment intensification. There is, however, a diabetes management protocol that has been developed but is not part of the electronic health records at this institution.

Currently, I have to pick up my phone, get on my coronary app and then put all the information. So, you could see where a tool like this that is incorporating the coronary risk score would be quite helpful built within Cerner. If it could even populate the data that we have with more recent blood pressure, that would be even more useful.

Primary care physician, male

Ideally, it would be something that I could just turn the computer monitor and show the patient, saying “Okay, well, this is why I'm recommending it. Your A1C is 5.9, up from 5.6 last year. Your cholesterol is up, your weight is up. So, this gives you a 17% chance of diabetes in the next two years. And these are the steps that we recommend...

Primary care physician, male

#### Diagnostic-Supported CDSS

Respondents noted a need for an CDSS that would consider diagnostics, such as relevant patient data and lab results. In addition, respondents indicated it would be useful to have access to clinically meaningful trends and track risk scores for complications. A majority of respondents manually calculate various risk scores, such as the 10-year Atherosclerotic Cardiovascular Disease (ASCVD) risk; therefore, embedding such a calculator in the CDSS could increase efficiency and reduce error.

If we had a good algorithm based on BMI and any potential risk factors that was easy to follow and implement, with good handouts and appropriate referral or community resources there, and if we could collate that information, I think it could be helpful.

Primary care physician, male

The main risk calculator that I use is the 10-year cardiovascular risk when I'm trying to decide if somebody should be on a statin. I just have on my phone and I just pull it up when I get their [patients’] labs back and plug in the numbers. Risk calculator would be helpful to have [embedded] in the computer as well.

Primary care physician, female

#### Incorporating Evidence-Based Practice

Respondents thought that having a CDSS that incorporates evidence-based clinical guidelines for management of obesity or overweight, both medical and behavioral, and that provides intervention recommendations would standardize and streamline the care provided and interventions suggested to their patients. The general idea was that such a system might help assist with managing patients with required tests, follow-up appointments, and preventive care.

I wouldn't say that I could speak for the whole clinic, I may just not be getting something that everyone else is doing. But we would be very open to having a tool that brings a standardization and also makes sure you're not overlooking anything and following a best practice guideline on initial management, and then also routine follow up.

Internist, female

I think what would be most beneficial is having suggested treatment plans. It would help me to know that I am on the right track if I had a treatment plan that was suggested based upon their [patient] other chronic co-morbidities and their current A1C results or current blood sugar trends.

Certified registered nurse practitioner, female

#### Ability to Have Resources to Make Referrals and Educate Patients

Respondents expressed a strong interest in information about accessible and affordable resources in the local community for patients struggling with overweight or obesity. They believed that it would enhance patient engagement and motivate patients toward behavior change. In addition, several respondents suggested that, if the CDSS had the means to efficiently provide appropriate educational materials to patients, it could improve the patient’s participation in their own care:

...having good patient-friendly handout material that was easy to attach in the patient portal in terms of a new diet, recommendations based on ADA or other more popular diets like the DASH (Mediterranean Diet) or other types of things based on the patient's history. I do think that would be helpful.

Primary care physician, female

Maybe [CDSS] gives you an option that you can click on, like option A “Would this person be interested in nutritional counseling?”, option B “Do you want to print this list of printouts to give them and present to them during your clinic visit?”, or “Would they want a referral to weight loss clinic?” I mean, it would be awesome if we had some way to refer people to some community resource near them where they could be contacted and offered some kind of like exercise class or a way to get into a walk group or something.

Certified registered nurse practitioner, female

## Discussion

### Principal Findings

In this study, we sought to understand and capture user requirements for a system that evaluates the stage and severity of cardiometabolic disease that would be incorporated into a CDSS. The idea to involve intended users early in the design process is well supported in the literature [26] and results in aligning user expectations with the resulting functionality [27]. Currently, the PCPs’ approach to management of overweight and obesity largely focuses on treating comorbidities and counseling lifestyle modifications such as diet and exercise. There is limited use of medications to combat overweight and obesity. Although there are existing guidelines for obesity treatment [28] and related cardiometabolic conditions such as diabetes [29] and hypertension [30], our respondents were not consistently using them for diagnosis or treatment purposes.

### Comparison With Prior Work

Findings from this study are consistent with a growing body of literature on how PCPs manage overweight and obesity, as well as on what CDSS features increase likelihood of its uptake. Turner et al [31] found, among a nationally representative sample of active health care providers, that (1) knowledge of physical activity and dietary guidelines was limited and (2) understanding of the appropriate initiation, intensity, and duration of pharmacotherapy was often inconsistent with evidence-based guidelines. Another study found that PCPs were least likely to say they would prescribe medication or refer a patient to counseling [32]. Regarding CDSS features, a systematic review by Groenhof et al [33] found that design and usability were important drivers behind the success, noting that information should be displayed all at once and at one glance. In addition, the lack of insight into the automated computation and source of information decreased user satisfaction. Further, the most recent systematic review by Kouri et al [34] identified important CDSS features that significantly predict uptake, such as averting the need for provider data entry by mining patient data from within electronic health record systems to inform CDSS.

### Implication for Practice

An important consideration would be providing easy access to the latest evidence-based clinical standards and protocols by embedding them in the CDSS. As a first step, CDSS could include measurement tools to perform a diagnostic evaluation based on evidence-based guidelines. If currently only BMI is considered, a more comprehensive evaluation must include additional measures such as measurement of waist circumference. Further, for the treatment, the physicians should be able to obtain clinical decision support by using CDSS to analyze pertinent information about the patient’s current clinical condition, including information about medication, lab results, and treatment compliance. Given support for the CDSS among our sample, we propose a design of a CDSS that provides suggestions for treating the primary and augmenting medications with explanations. For the purposes of follow-up, the CDSS should have reminders to ensure the important considerations are not overlooked. Moreover, it could also recommend and display when the patient should return for a visit. All entries should be automatically stored, providing electronic documentation and record keeping, thus providing access to complete patient information. Overall, information about a patient’s demographic characteristics and other clinical records should be accessible by a single click.

### Implications for Development

**Figure 2:**
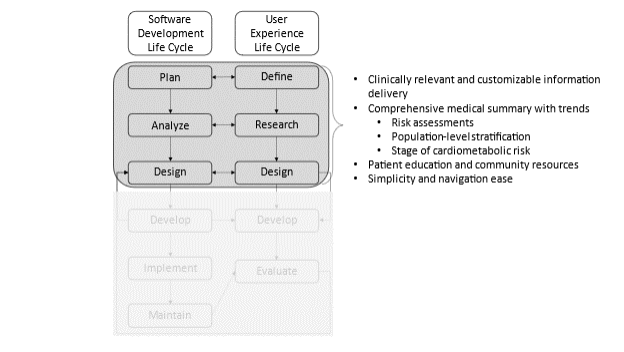
The conceptual framework with the findings as high-level categories.

Results from this study were used to better understand user requirements within a parallel system analysis and design framework (see Figure 1), the importance of which was to ensure the voice of the user was adequately and accurately represented [20]. In this phase, we present the conceptual framework with the findings applied as high-level categories (see Figure 2). These categories and the details behind them as presented throughout this study will be used to inform the evaluation.

### Limitations

There are several limitations of this study. First, the 10 participants, predominantly non-Hispanic White (9/10, 90%), represented PCPs at a large academic center in the southeast. A small homogenous sample size could potentially limit the generalizability of our findings, and we recognize the need to add breadth and depth to this participant sample as development ensues. In addition, the strength of the study was our consideration of assuring intercoder reliability. Thus, we feel confident that we are correctly representing the voices of our participants.

### Conclusion

Implementation of a CMDS system in the form of a CDSS could be used as a risk assessment tool that also provides risk-based and evidence-based treatment or program recommendations to better manage overweight and obesity and prevent diabetes. Results from this study provide unique insight to developers and researchers to identify areas for design optimization for improved end user experience to ensure successful adoption of the CDSS.

## Appendix

Multimedia Appendix 1 COREQ checklist.

Multimedia Appendix 2 Interview guide.

## Abbreviations

ASCVD: Atherosclerotic Cardiovascular Disease
CDSS: computerized clinical decision support system
CMDS: cardiometabolic disease staging
COREQ: consolidated criteria for reporting qualitative studies
HDL-C: high-density lipoprotein cholesterol
PCP: primary care provider
UAB: University of Alabama at Birmingham
