# Supplementary material for: Exploring a Need for a Cardiometabolic Disease Staging System as a Computerized Clinical Decision Support Tool: Qualitative Study
Source: JMIR Form Res. 2022 Jul 1;6(7):e37456. doi: 10.2196/37456 (PMC9288101; doi:10.2196/37456)
Supplement: Multimedia Appendix 2 [file formative_v6i7e37456_app2.docx]

**Multimedia Appendix 2. Interview guide.**

**Focus Group and One-on-One Interview Script**

**Start Recording**

**INTRODUCTION**

**Interviewer:** Before getting into specific questions, I’d like to start by giving you a brief overview of the project. The purpose of these focus groups and interviews are to discuss your preferences around how to best support your diagnoses and treatment of overweight, obesity and facilitate prevention and management of diabetes and cardiovascular disease risk by using a clinical decision support system. The interview will last around 30 to 45 minutes. With your permission, today’s interview will be recorded, primarily so we can capture your insights accurately.

The information that we gather is confidential and no one else outside of the evaluation team can access the specific information you provide. It is our intention, however, to summarize the information provided by you and other people for the purposes of identifying the most appropriate implementation strategies of clinical decision support system into a clinician’s workflow to help and not hinder their use and increase efficiency.

Your participation in today’s interview is entirely voluntary. You may also change your mind later and stop participating at any time, even if you agreed to participate earlier. Likewise, if you do not want to answer any of the questions during the interview, you can say so and I will move on to the next question. Do you have any questions before we begin?

So, before we get into specific questions:

1. Do I have your consent to participate in today’s interview?
   1. If no, “thank you for your time”.
   2. If yes, do I have your permission to record the interview?
      1. If yes, move on to Background questions.
      2. If no, turn off recorder.

**BACKGROUND**

1. **Please tell us about your role in the clinic.**
   1. **Probes: how long have you been practicing and how long have you been working in your current clinic?**
   2. **What kind of patients do you see? What is the most prevalent “condition” (obesity, overweight, diabetes or any other metabolic condition)?**

.

**Question 1:** **First we would like to understand your workflow and current practice patterns. Please describe your approach to medical management of overweight and obesity, to prevent and manage the risk of diabetes and cardiovascular disease.**

1. For example, a person with a BMI of 33 comes into your office for their first visit. How do you approach the topic of their obesity and risk factors for developing cardiometabolic conditions, such as diabetes?
2. What do you use as a diagnosis measure for overweight and obesity (BMI, waist circumference)? What diagnoses guidelines do you follow?
3. What factors do you consider when you are developing a treatment plan for a person with overweight, or obesity, or pre-diabetes? Are those factors different for different population groups, e.g. low-income, diet problems? Do you follow specific treatment guidelines?
4. Do you prescribe any pharmacological intervention?
5. Do you prescribe or refer patients to any specialists that provide intense lifestyle modification?
6. Please describe any printed materials you share with them. Do you show them exercise options, nutrition, etc. charts? Referrals, counseling, diets to follow?
7. Do you have a multidisciplinary team to coordinate the care?
8. How much do you interact with the computer and receive decision support during a patient visit?
9. How does the current initiative of BMI screening and follow-up, which is based on CMS requirements, influence your workflow and obesity related care?

**Question 2:** Does your clinic currently have any clinical decisions support system (CDSS) in place for patients with obesity, overweight to reduce or manage the risk of diabetes or CVD?

- - If yes:
    1. Can you tell me about this system?
    2. What is the potential usage for CDSS? (disease prevention, diagnosis, therapy, allergy alerts, follow-up?)
    3. What advantages and disadvantages do you see in the existing CDSS?
  - If no:
    1. Is there any particular reason why your clinic has not pursued these types of systems?
    2. What advantages do you think the CDSS may provide over your existing approach to manage patients with cardiometabolic conditions?
    3. What disadvantages CDSS provide over your existing approach to management of these conditions?

**Question 3:** Based on your knowledge of the CDSS, what kinds of changes, if any, do you think you may need to make to the CDSS to make it work effectively in your clinic?

- - Do you think you have enough information about the patient? (medication lists, lab results, etc.?)
  - What would be “right information” and “too much information” to make a decision?
  - Are you able to share data if necessary?
  - What about user computer interaction? Would you like to customize certain features, like alerts/reminders?
  - Are there any built-in definitions of risk measures (do you think they are needed)?
  - How simple and easy is it to use?
  - Would you find a CDSS that incorporates diabetes and CVD risk assessment, and based on the risk then outlines a treatment plan helpful assuming there is ability to customize the plan?

**Question 4:** From the perspective of clinic personnel, what do you think will be the primary barriers to using the CDSS?

- - Probe: Do you feel time pressure? Or IT skills?
  - Do you think by using the computer to access information, you may threaten the current-patient relationship?
  - Would you consider it as an extra workload due to excessive reminders?
  - Issues of compatibility and updating, problems with several poorly interacting computer programs? Poor wi-fi connection?

**Interviewer:** Patient portals are a used to improve quality of care and access to care by enabling patients to review some information from their medical records and to view other information such as treatment options and referrals.

**Question 5:** What are your perceptions of the use of the patient portal to communicate information about management of overweight, obesity, to reduce risk of diabetes or CVD incidence when they have left the office?

1. Would you suggest that we use the patient portal to communicate information? Why or Why not?
2. What information would you suggest presenting in the patient portal?

**Question 6:** Is there anything that we haven’t discussed today that you think would be helpful for us to know when thinking about implementing the CDSS in your clinic?

**CONCLUSION**

Thank you again for taking time out of your busy schedule to talk with us today.

**End recording.**
